# A *de novo* 10.79 Mb interstitial deletion at 2q13q14.2 involving *PAX8* causing hypothyroidism and mullerian agenesis: a novel case report and literature review

**DOI:** 10.1186/s13039-014-0085-4

**Published:** 2014-11-26

**Authors:** Deqiong Ma, Robert Marion, Netra Prasad Punjabi, Elaine Pereira, Joy Samanich, Chhavi Agarwal, Jianli Li, Chih-Kang Huang, K H Ramesh, Linda A Cannizzaro, Rizwan Naeem

**Affiliations:** Molecular Pathology and Cytogenetics Lab, Department of Pathology, Montefiore Medical Center, Albert Einstein College of Medicine, 1635 Poplar Street, Bronx, NY 10461 USA; Department of Pediatrics, Children’s Hospital at Montefiore, Bronx, NY USA

**Keywords:** Interstitial deletion at 2q13q14.2, Hypothyroidism, Mullerian agenesis

## Abstract

Reports of interstitial deletions involving proximal long arm of chromosome 2 are limited. Based on early chromosomal analysis studies, the phenotypic consequence of deletions at the ancestral chromosome fusion site at chromosome 2q13q14.1 remains unclear. A recurrent 1.71 Mb deletion at 2q13 has recently been proposed as a new genomic disorder, associated with an increased risk of intellectual disability and craniofacial dysmorphism. Herein, we report the case of a 12 year-old girl with unique clinical features including global developmental delay, mullerian agenesis, and hypothyroidism associated with a normal size and position of the thyroid gland, as well as negative thyroid antibodies. Microarray-based comparative genomic hybridization study revealed a *de novo* 10.79 Mb deletion at 2q13q14.2 (111,548,932–122,336,492), which involves more than 88 UCSC genes, 38 of which are OMIM genes, 7 of which are disease-causing and 3 of which (including *GLI2*, *IL1B* and *PAX8*) show a dominant inheritance pattern.. Interestingly, *PAX8* (chr2:113,973,574–114,036,498), a member of the paired-box gene family, is essential for the formation of thyroxine-producing follicular cells. Autosomal dominant transmission of congenital thyroid hypoplasia due to loss-of-function mutation of *PAX8* suggests a possible haploinsufficiency effect. Additionally, *PAX8* is also expressed in the tissue primordia that form both the mullerian duct derivatives and the upper urinary tracts. A recent study has associated a novel *PAX8* mutation with a severe form of hypothyroidism and abnormalities in the urogenital tract. Taken together, the unique clinical manifestation seen in this patient could be attributed to the heterozygous deletion of *PAX8* gene. A prospective investigation is merited to fully evaluate the pathogenic effect of the interstitial deletion of 2q13q14.2.

## Background

Recombination is the main cause of genetic diversity. Errors in recombination lead to a variety of chromosomal abnormalities [1]. Through the application of microarray comparative genomic hybridization (aCGH) technology, identification of both recurrent and atypical genomic alterations, such as copy number variants becomes a conventional approach for discovering new genomic disorders and mapping dosage sensitive genes [2]. In the era of personalized genome medicine, this technique allows the molecular characterization of the breakpoints at a finer scale [3,4], which ultimately will improve the delivery of healthcare for individuals affected with these abnormalities.

The proximal long arm of chromosome 2 contains the ancestral chromosome fusion site at 2q13q14.1 [5]. Studies examining genotype-phenotype correlations at this site, are limited. For instance, based on early chromosomal and FISH studies, the phenotypic consequence of 2q13q14.1 deletion is not well known [6-9]. Recently, an aCGH-based study proposed a 1.71 Mb recurrent genomic disorder at 2q13 (111392197–113102594) [10], which is linked to an increased risk of intellectual disability and craniofacial dysmorphism; a wide range of clinical presentations was seen in individuals found to be affected with this deletion. Studies of atypical deletions in this region have furthered the understanding of genotype-phenotype correlation through cases with distinct clinical features including pontine tegmental cap dysplasia involving *NPHP1* gene (chr2:110862477–110959127) [11], gastrointestinal tumours involving *BUB1* gene (chr2:111399043–113102735) [12], congenital heart defect involving *FBLN7 and TMEM87B* gene (chr2:111442130–113937615) [13] and chronic cutaneous pustulosis, a newly recognized disease caused by 175 kb deletion involving *IL1RN* and five adjacent genes (*IL1F9, IL1F6, IL1F8, IL36RN, and IL1F10*) [14].

Herein we report the case of a 12-year-old girl with multiple congenital and developmental anomalies, who was found to be carrying a *de novo* 10.79 Mb interstitial deletion at 2q13q14.2 (111,548,932–122,336,492), which involves *PAX8* gene. Novel features found in this patient include mullerian agenesis (also known as Mayer-Rokitansky-Küster-Hauser syndrome, or the Rokitansky malformation sequence), hypothyroidism and global developmental delay.

## Case presentation

### Medical history

C.N was the second child born to healthy, non-consanguineous parents; her mother was 28 years of age at the time of her birth. Family history was non-contributory. The pregnancy was complicated by oligohydramnios and reduced fetal movements. Amniocentesis was normal. She was born via an uncomplicated elective Cesarean section due to a previous Cesarean section in a hospital in the Dominican Republic at 37 weeks of gestation, with a birth weight of 6.75 lbs. Apgar scores were 9 at one minute and 10 at 5 minutes. Neonatal course was uncomplicated and she was discharged from the well-baby nursery on the fourth day of life (the nursery stay elongated because of the mother’s caesarean section).

Signs of developmental delay were apparent to the parents by 2–4 months. CN began sitting independently at 12 months and walked at two and half years of age; because of these delays, she was referred for early intervention services and received occupational, physical and speech therapies, each two times per week. During her first two years, scoliosis was diagnosed, and she experienced recurrent episodes of otitis media requiring the placement of tympanostomy tubes.

CN came to the US with her family at age 2. During childhood, she was noted to have short stature and some craniofacial dysmorphic features. A preliminary work-up revealed an elevated level of thyroid stimulating hormone (TSH), with thyroid hormone (T4) levels in the normal range. On the basis of these findings, she was referred for evaluation to endocrinology.

At endocrine clinic, initial laboratory investigations revealed normal chemistry, liver function tests, celiac screen, and IGF-1 level. Testing confirmed an elevated TSH (9.610 uIU/ml [0.70–5.70]) with a low normal free T4 level (0.92 ng/dl [0.80–1.90]) and negative thyroid antibodies. Her gonadotropins were normal with a delayed bone age. Initial pelvic sonogram showed normal prepubertal uterus with no visualization of ovaries. Cytogenetic analysis (metaphase banded chromosomes) was interpreted as normal. The patient was begun on thyroid hormone supplementation.

CN was followed over several months for short stature and maintained on 50mcg synthroid. Repeat thyroid function tests revealed that with treatment, she was in the euthyroid range. Because of severe right lower quadrant pain, she was brought to the emergency department for evaluation. Work-up revealed torsion of her right fallopian tube, a blind vaginal pouch, and a prepubertal hemiuterus with normal-appearing kidneys. She underwent emergency surgery with right salpingectomy and hemiuterus resection. A growth hormone stimulation test with clonidine and arginine was done 1 month thereafter with normal cortisol and growth hormone response.

Because of her multiple anomalies, associated with intellectual disabilities and short stature, CN was referred to genetics clinic for evaluation.

### Physical examination

**Figure 1:**
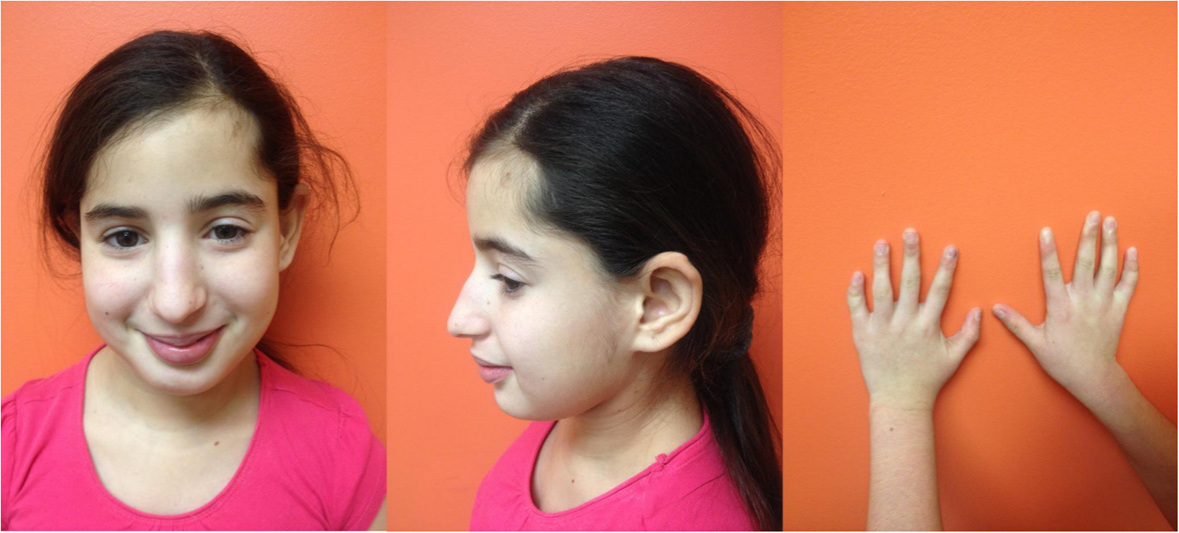
**Patient images.** Facial anomalies limited to slight frontal bossing, deeply set eyes, long nose with a bulbous tip, large protruding ears, and long eyelashes and clinodactyly.

Examination in genetics clinic at 12 years of age showed a weight of 34.8 kg (8^th^ percentile), height of 139.8 cm (<3^rd^ percentile), and an occipitofrontal circumference (OFC) of 51.3 cm (50^th^ percentile). Facial features included slight frontal bossing, deeply set eyes, long nose with a bulbous tip, large protruding ears, and long eyelashes (Figure 1). She also had hypotonia, clinodactyly of the fifth fingers bilaterally, hallux valgus, cubitus valgus, pes planus and hypoplastic palmar crease pattern bilaterally without apparent dermatoglyphic abnormalities. Skin examination revealed no lesions. She demonstrated Tanner 4 breast and pubic hair development. Mild scoliosis was appreciated.

### Thyroid sonography

A sonogram of the thyroid was performed assessing gray scale appearance and color Doppler flow. The right lobe measures as 1.4 × 2.4 × 0.7 cm. The left lobe measures as 1.3 × 3 × 0.9 cm. The thyroid gland was homogeneous in echo texture. There is no discrete mass or cyst and normal vascularity was noted. The isthmus measures as 0.2 cm in thickness. No isthmus nodule is seen.

### Chromosome analysis

**Figure 2:**
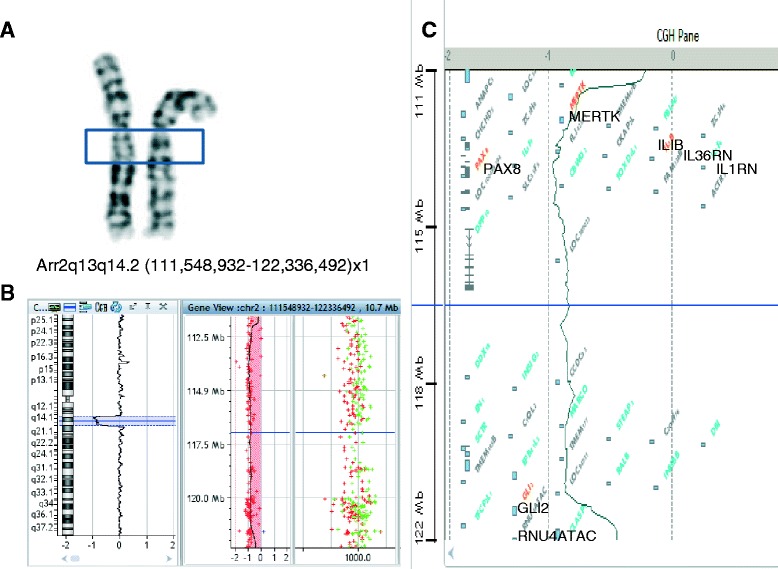
**Chromosome and aCGH findings. A**: chromosome analysis; **B**: Array-CGH profile of chromosome 2; **C**: OMIM morbid genes within the deletion regions.

Standard methods were followed while performing culturing, harvesting of peripheral blood cells and chromosome banding. GTG-banded chromosomes were studied and the karyotype was interpreted according to the International System for Human Cytogenetic Nomenclature (ISCN 2013). Retrospective cytogenetic analysis revealed a band deletion at 2q13q14 (Figure 2A) at the 550 band level.

### Microarray-Comparative Genomic Hybridization (aCGH)

aCGH was carried out using the Agilent Human Genome CGH Microarray Kit (Agilent Technologies, Santa Clara, California, USA). DNA was labeled using Agilent Suretag DNA Labeling Kit, and applied to 44 K oligonucleotide arrays. Image quantification, array quality control and aberration detection were performed using Feature Extraction and Cytogenomics 2.7 software packages [ADM-2 algorithm employed] (Agilent, USA). A 10.79 Mb deletion at 2q13-14.2 (111,548,932–122,336,492) [hg19] (Figure 2B), which is consistent with the result from cytogenetic analysis (Figure 2A). Parental aCGH studies were normal, indicating that the deletion in the proband occurred *de novo*.

### Discussion

As mentioned above, only a few cases with a proximal interstitial deletion at chromosome 2q13q14 have been previously reported. A review of these cases reveals contradictory phenotytpic findings. Individuals with this deletion demonstrate a wide spectrum of clinical features ranging from no obvious abnormalities, through mildly dysmorphic feature to severe intellectual disabilities and multiple congenital malformations [6-9]. Since in the past, breakpoint mapping has been based on karyotype and FISH results, direct comparisons with our case are not possible. A comprehensive search in our internal, DECIPHER and ISCA databases failed to identify any cases carrying a deletion that overlapped with the *PAX8* gene or in which a similar clinical presentation to the case being reported here was identified. Thus, this is the first report of a patient carrying a 10.79 Mb deletion at 2q13q14.2 (111,548,932–122,336,492) in which unique clinical features, including global intellectual disability, mullerian agenesis, and hypothyroidism with a normal size and position of the thyroid, have been identified. The molecular characterization of the large deletion at 2q13q14.2 provides a clue for the etiology of the hypothyroidism and mullerian agenesis and potential strategies for future surveillance.

The *de novo* deletion identified in patient CN overlaps with the previously reported genomic deletion at chromosome 2q13 (111392197–113102594) [10]. The currently described deletion involves more than 88 UCSC genes, 38 of which are OMIM genes and seven of which are OMIM morbid (Figure 2C). Among the seven disease-causing genes, a recessive mode of inheritance has been reported for *MERTK* (Proto-oncogene tyrosine kinase MER), *IL36RN* (Interleukin 36 receptor antagonist), *RNU4ATAC* (Small nuclear RNA 4atac) and *IL1RN* (Interleukin 1 receptor antagonist), while an autosomal dominant pattern is documented for *PAX8* (Paired box gene 8), *GLI2* (GLI-Kruppel family member 2) and *IL1B* (Interleukin 1-Beta). Loss-of-function mutations inherited in a dominant fashion have been documented in OMIM for *PAX8* and *GL12*, suggesting a possible haploinsufficency of their proteins.

Congenital hypothyroidism (CH) is a condition that affects infants from birth (congenital) and results from a partial or complete loss of thyroid function. The most common congenital endocrine disorder, it has been noted to affects 1 in 2700 newborns, according to a recent European survey [15]. In between 80 and 85 percent of cases, the thyroid gland is absent, abnormally located, or hypoplastic. In the remaining cases, a normal-sized or enlarged thyroid gland is present, but production of thyroid hormones is decreased or absent [16]. The candidate genes associated with this genetically heterogeneous disorder are classified into two main groups: those causing thyroid gland dysgenesis and those causing dyshormonogenesis. Genes associated with thyroid gland dysgenesis include the TSH receptor in non-syndromic CH, and Gsalpha and the thyroid transcription factors (*TTF-1, TTF-2,* and *PAX8*), associated with different complex syndromes that include CH [17]. However, thyroid morphology of those mutation carriers is variable (i.e. normal to severe hypoplasia). So far, the only systematic genetic screening of patients with CH is a study from Japan, in which 102 newborns with CH, who are either with or without thyroid dysgenesis (TD) revealed that *PAX8* mutations are the leading cause with a prevalence of 2% in the cohort among the mutations of four transcription factors including *PAX8*, *NKX2-1* [encoding thyroid transcription factor (TTF)-1], *FOXE1* (encoding TTF-2), and *NKX2-5*, [18].

*PAX8* is a member of the paired-box gene family and expressed in embryogenesis of the thyroid, Mullerian and renal/upper urinary tracts, as well as in carcinomas from each of these sites [19,20]. It regulates the expression of genes encoding thyroglobulin (TG), thyroid peroxidase (TPO), and the sodium-iodide symporter (NIS) by binding to the promoter regions through its 128-amino acid paired domain [21,22], which is essential for the formation of thyroxine-producing follicular cells. Its point mutations and small indels are recognized causes of CH mainly through a loss-of-function effect [23]; however, some cases can present with a normal sized thyroid gland [24], which is the case for our patient.

Additionally, although it usually occurs in isolation, CH is also seen as a part of multiple congenital malformation syndromes, predominantly associated with congenital heart disease, neural tube defects and dysmorphic features [25,26]. *PAX8*-specific extrathyroid malformation syndrome has recently been reported in a multi-generation family in which six members were found to carry a novel heterozygous point mutation (c.74C > G). Features of this condition combine a severe form of congenital hypothyroidism with urogenital malformations, including incomplete horseshoe kidney and hydrocele present in females as well as undescended testicles and ureterocele in males [27]. For our patient in whom a complete one-copy deletion of *PAX8* was identified, extrathyroid malformations of the urogenital tract included Rokitansky sequence (mullerian agenesis) associated with torsion of the right fallopian tube. No obvious malformation was found in the kidneys.

*GL12* and *IL1B* are two other genes in the deleted region that demonstrate autosomal dominant modes of inheritance. The *GLI2* transcription factor is a major effector protein of the sonic hedgehog pathway and has been reported to play a key role in pituitary development [28]. *M*utations in this gene are associated with anterior pituitary hypoplasia and, frequently, ectopy of the posterior lobe of the pituitary results [29]. Genomic *GLI2* aberrations that mainly result in truncated proteins have been reported to cause holoprosencephaly or holoprosencephaly-like features, sometimes associated with hypopituitarism [30]. *GLI2* mutations with proven pathogenic impact have all been either heterozygous of nonsense orframeshift mutations, all leading to truncated proteins lacking a functional C-terminal transactivation domain. Loss of function has been demonstrated for some of them by distinct in vitro approaches, but experimental reasoning is so far limited to the mutations identified by Roessler et al. [31,32]. In general, the phenotype of *GLI2* mutations is variable, and penetrance is incomplete as further suggested by a recent family segregation study, in which the proband carried a 1.3 Mb deletion encompassing *GLI2* gene [33]. Our patient apparently does not demonstrate features of either holoprosencephalyor hypopituitarism..

The human interleukin 1-Beta (*IL1B*) gene plays an important role in responding to microbial invasion, inflammation, immunologic reactions, and tissue repair [34]. Our patient presented with recurrent ear infections, which could be related to lack of *IL1B*. Loughlin, et al. describes IL-1 gene cluster at 2q13 as being the main catabolic cytokine of the joint with osteoarthritis, and can stimulate synthesis of proteinases, which can cause the breakdown of cartilage extracellular matrix proteins [35]. A prospective surveillance may be necessary to evaluate the possible pathogenic effect via haploinsufficiency of *IL1B* in the patient.

## Conclusion

This case is an example of a commonly-occurring problem: a child with multiple congenital anomalies in whom a unifying diagnosis is not obvious. Although the importance of using aCGH as a first-tier diagnostic tool in individuals with unexplained developmental delay and unusual phenotypes cannot be overstated, use of aCGH to identify a CNV in such a patient would provide the opportunity for early diagnosis, providing the possibility of early treatment and genetic counseling for the family. In the present case, the very unique manifestation of the non-autoimmune and possible congenital hypothyroidism with a normal size and position of thyroid and mullerian agenesis could be attributed to the heterozygous deletion of *PAX8* gene in this case. A prospective investigation is merited to fully evaluate the pathogenic effect of the interstitial deletion of 2q13q14.2.

## Consent

A written informed consent was obtained from the parents of the patients for publication of this report and the accompanying images. A copy of the written consent is available for review by the Editor-in-Chief of this journal.
